# Integrating Artificial Intelligence in Nursing Practice With Decubitus Risk Prediction Alerts: A Pilot Process Evaluation

**DOI:** 10.1111/jocn.70212

**Published:** 2026-01-20

**Authors:** Denise Spoon, Annemarie de Vroed, Steffen Greup, Monique van Dijk, Erwin Ista

**Affiliations:** ^1^ Department of Internal Medicine, Division of Nursing Science Erasmus MC, Erasmus University Medical Centre Rotterdam the Netherlands; ^2^ Department of Quality and Patient Care Erasmus MC, Erasmus University Medical Centre Rotterdam the Netherlands; ^3^ Department of Data & Analytics Erasmus MC, Erasmus University Medical Centre Rotterdam the Netherlands; ^4^ Department of Neonatal and Pediatric Intensive Care, Division of Pediatric Intensive Care Erasmus MC – Sophia Children's Hospital, Erasmus University Medical Centre Rotterdam the Netherlands

**Keywords:** clinical decision‐making, fundamentals of care, hospital, implementation, information technology, nurses, nurse's responsibilities, pressure ulcer, risk assessment

## Abstract

**Aims:**

To evaluate the acceptability and feasibility among nurses of Decubitus Risk Prediction Alerts based on Artificial Intelligence (DRAAI), and to assess the feasibility of the implementation plan.

**Design:**

A process evaluation of a pilot implementation study using mixed methods.

**Methods:**

Acceptability and feasibility of DRAAI among nurses from three general wards in a university hospital was assessed via questionnaire. The tailored implementation plan included thirteen strategies distributed over six domains, such as facilitation, continuous evaluation, and educational sessions. Adaptations, acceptability, and feasibility were recorded in field notes.

**Results:**

Fifty‐five nurses completed the questionnaire and valued DRAAI's predictions, believing these could contribute to pressure ulcer (PU) prevention. Some initially faced challenges distinguishing between PU risk and PU detection. Most found it feasible to integrate DRAAI into their workflow. Adaptations included adding PU preventive measures to educational sessions and sharing frequently asked questions and answers. Overall, implementation efforts were feasible. DRAAI generated PU risk predictions for 428 unique admitted patients; 128 (30%) patients received at least one at‐risk prediction. Regarding fidelity, nearly 80% (101/128) of at‐risk predictions were followed by a nursing care plan.

**Conclusion:**

Ongoing involvement and clear communication were crucial for successfully integrating AI into nursing workflows. Although some nurses were concerned that DRAAI might miss at‐risk patients, they continued to independently identify at‐risk patients.

**Implications for the Profession and/or Patient Care:**

Implementation of DRAAI served as a prompt for nurses to focus more on PU prevention. While DRAAI shows promise in improving PU prevention, future research is needed to evaluate its clinical impact.

**Impact:**

Addressed the challenge of identifying patients at risk for developing pressure ulcers. Demonstrated feasibility and acceptability of implementing AI in clinical practice. Highlighted the need for ongoing support and communication for successful implementation.

**Patient Contribution:**

None.

**Reporting Method:**

Standard for Reporting Implementation Studies (StaRI).

## What Does This Paper Contribute to the Wider Global Clinical Community?

- Demonstrates the feasibility of integrating AI in clinical practice for PU prevention.
- Offers valuable insights into the implementation process and key success factors.
- Highlights AI's potential to optimize nursing workflows and improve patient care outcomes.

## 1 Introduction

Pressure ulcers (PU) are a source of harm and discomfort for hospitalised patients, often resulting in prolonged hospital stays, increased healthcare costs, (Goodall et al. 2020; Krupová et al. 2025) and higher workload for healthcare professionals (Reis et al. 2023). The causes of PU are multifactorial, with key patient‐related factors including obesity, being underweight, smoking, diabetes mellitus, and immobility due to illness (Chung et al. 2022; Coleman et al. 2013; Nieto‐García et al. 2022). Treatment‐related factors such as anaemia, corticosteroid treatments, or chemotherapy further elevate the risk (Chung et al. 2022). Additionally, surface‐ or device‐related factors, such as suboptimal mattresses, seating cushions, and medical devices like catheters and drains, can also contribute to PU development (Shi et al. 2021). These fluctuating multifactorial risk factors during hospital stays create challenges for nurses in promptly identify patients at‐risk for developing PU.

Validated PU risk assessment tools include the 11‐item Waterlow Score (The Waterlow Score 2005; Charalambous et al. 2018) and the 6‐item Braden scale (Bergstrom et al. 1987; Huang et al. 2021). These tools incorporate several of the previously mentioned risk factors. However, their measurement properties are not optimal, with sensitivity ranging from 46% to 82%, and specificity ranging from 27% to 67% (Gould et al. 2004; Pancorbo‐Hidalgo et al. 2006; Kottner et al. 2009). Additionally, the multifactorial nature of PU risk factors leads to fluctuations in patients' risk level throughout their hospital stay.

The European Pressure Ulcer Advisory Panel (EPUAP) guidelines recommend regular PU risk assessments to address these fluctuation (European Pressure Ulcer Advisory Panel, National Pressure Injury Advisory Panel, and Pan Pacific Pressure Injury Alliance 2019). However, performing regular risk assessments can contribute to a sense of administrative burden among nurses. This is particularly evident when clinical reasoning suggests that a patient is not at risk, making repeated risk assessments feel unnecessary (Zegers et al. 2022).

Research demonstrates the potential of machine learning models to predict PU risk (Jiang et al. 2021; Hu et al. 2020; Anderson et al. 2021; Pei et al. 2023). While these models have shown outstanding performance, evidence concerning their clinical value in practice remain limited (Wang et al. 2023). In our search for a fitting prediction model, we were unable to find one which was directly applicable to our setting for intended use, general wards of a tertiary university hospital. Most models included only patients scheduled for surgery or used predictors that are typical for a specific culture like betel nut chewing (Hu et al. 2020; Anderson et al. 2021). This posed challenges due to the lack of available data that could be used in our context.

Considering the fluctuating risk of PU risk, a frequently updating risk prediction model can eliminate the need for repeated manual risk assessments. To address this, a team of wound care nurses, PU nurse champions, and data scientists developed an AI‐based PU prediction model called DRAAI. DRAAI, an acronym for Decubitus Risk Alert Artificial Intelligence, also means ‘turn’ in Dutch. The model enables daily predictions of PU risk relying on a logistic regression wit an L2 penalty and spline transformation applied to a limited set of predictors.

The rapid adoption of generative AI by the public, driven by advancements from industry, has outpaced academia in some respects. Nonetheless, the number of academic publications on AI has tripled from 88,000 in 2010 and 240,000 in 2022 (Maslej 2024). However, as Van de Sande et al. (2021) highlighted in a systematic review of AI‐applications in intensive care units, most AI models remain confined to the testing and prototyping phase; with only a small fraction being evaluated in real‐world clinical practice. Incorporating the principle of implementation science early in the development of AI has the potential to improve its adoption and integration into clinical workflows. Despite this potential, few studies have focused on using implementation science to facilitate the deployment of AI in healthcare settings (Finkelstein et al. 2024; Gama et al. 2022). To our knowledge, conducting a comprehensive mixed‐method process evaluation of the pilot implementation of an AI‐based risk prediction model like DRAAI is a novel contribution to this field.

In this study the main research question is: ‘How is the pilot‐implementation of DRAAI experienced and carried out in clinical nursing practice, and what adaptations are necessary to optimise its use in preventing pressure ulcers?’ This pilot implementation study focused on the following implementation outcomes acceptability, feasibility, dose, adaptations, fidelity, and timeliness.

## 2 The Study

### 2.1 Aims

This process evaluation aimed to evaluate acceptability and feasibility of the Decubitus Risk prediction Alert based on Artificial Intelligence (DRAAI) prediction model among nurses. The secondary aim was to evaluate the feasibility of the implementation plan by quantifying the implementation efforts and analysing nurses' responses to these efforts. The third aim was to examine fidelity to the risk predictions, which was defined as the degree to which nurses adhered to DRAAI's risk alerts. Additionally, the timeliness of the follow‐up actions based on these risk predictions was also assessed.

## 3 Methods

### 3.1 Design

This process evaluation of a mixed‐method pilot implementation study was conducted according to the principles of Moore et al. (2015).

### 3.2 Study Setting

The study was conducted between June and September 2023 in three general wards (Covid‐19, pulmonary, and internal medicine) at Erasmus MC, a tertiary university hospital in the Netherlands. These wards had 24–33 beds and 26–58 nurses. The latter represent the total number of nurses working on these wards, not the full time equivalent, and do not include nursing students. Managers' enthusiasm and their expressed need for a different approach to PU prevention were key factors in selecting these wards.

### 3.3 The Intervention—DRAAI

DRAAI, a clinical decision support system (CDSS), was developed using literature, expert input, and retrospective hospital data.

DRAAI was developed based on existing literature (Hu et al. 2020; Anderson et al. 2021; Park and Lee 2016), expert opinions, and retrospective analysis of PU patients within our hospital. It predicts PU risk using routinely collected electronic health record data (e.g., age, mobility, lab results). Its predictive performance (AUC 0.80–0.82) surpassed that of the Waterlow score (AUC 0.68–0.70) (Greup et al. 2024). Dozens of predictors were selected from routinely collected data in the electronic patient records, including open text, continuous, and categorical fields. Examples of these predictors include age, expected remaining length of stay, mobility level, prescribed medications, planned surgeries, and laboratory results such as albumin levels. DRAAI predictions were validated using nursing diagnoses of PU (Carpenito 2016), medical diagnoses, and PU observations recorded during prevalence measurements. These measurements are conducted three times a year according to hospital protocol, adhering to the EPUAP statement on PU prevalence monitoring (Defloor et al. 2005). The predictive performance of the final model, measured by the area under the ROC curve, exceeded the predictive performance of the Waterlow risk assessments (0.80–0.82 vs. 0.68–0.70). A detailed description of the development, validation, and calibration of DRAAI is provided in a separate publication (Greup et al. 2024).

DRAAI operates as a clinical decision support system (CDSS), designed ‘to enhance decision‐making in the clinical workflow’ by providing tools such as alerts, guidelines, order sets, templates, and summaries (Mills 2019). Utilising person‐specific data and reasoning mechanisms, a CDDS generates and presents valuable information to nurses (Garg et al. 2005).

**FIGURE 1:**
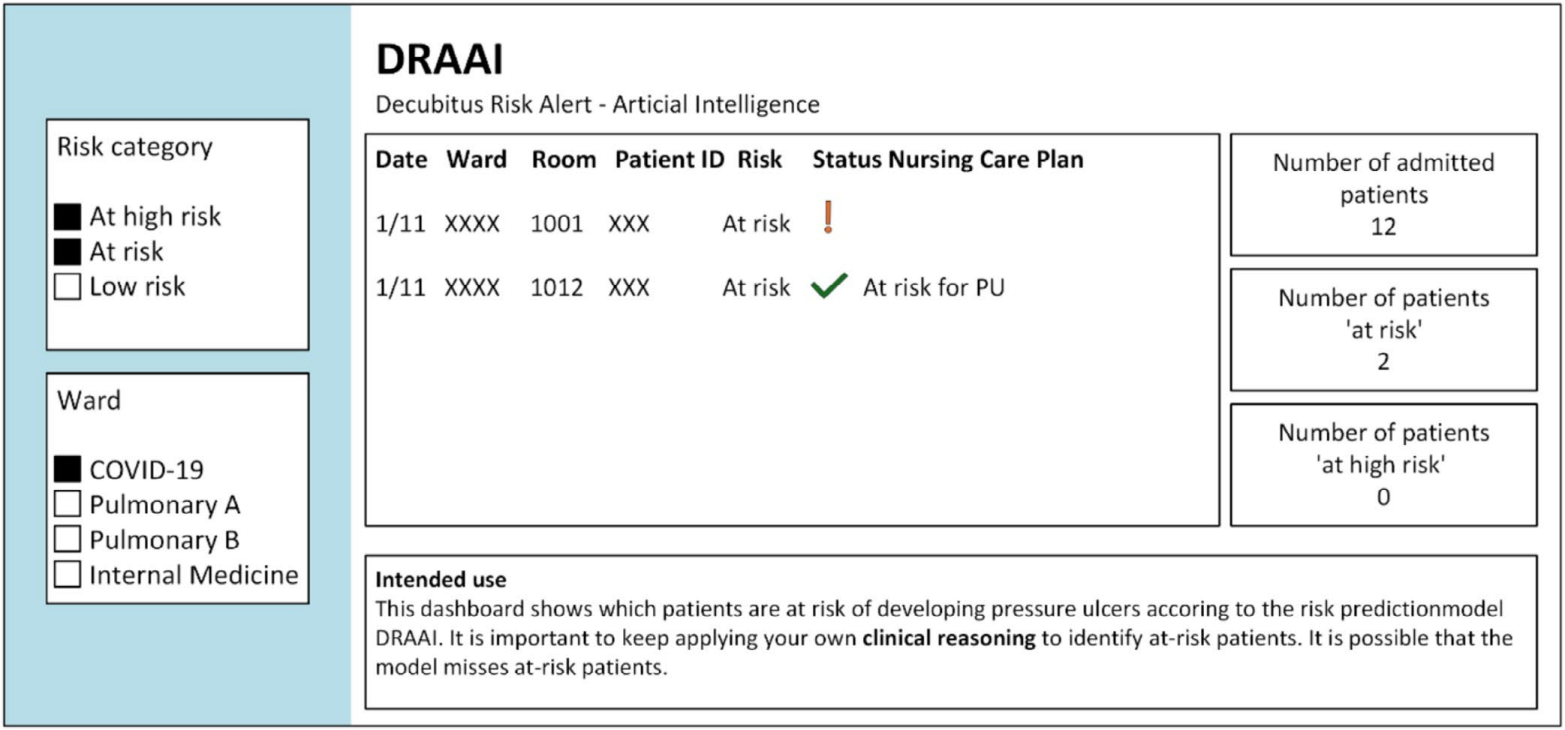
Visualisation of DRAAI's web application at the start of the trial, presenting at (high) risk patients from a specific ward with real‐time feedback on follow‐up actions of the predictions with the status of the nursing care plan (at risk of PU). [Colour figure can be viewed at wileyonlinelibrary.com]

DRAAI provides daily PU risk predictions (low, at‐risk, high) based on extracted risk predictors in the electronic patient files from the previous 24 h. At the time of the pilot study these predictions were available via a stand alone web application. Nurses were instructed to use these predictions—alongside clinical reasoning and, if needed, the Braden score (Bergstrom et al. 1987; Huang et al. 2021) —followed by creating nursing care plans for at‐risk patients in the electronic patient record. These plans include preventive measures and prompt daily evaluation. A feedback loop offers real‐time updates on care plan status. Figure 1 shows the DRAAI dashboard, as it appeared at the start of the pilot study. See Appendix S3 of this paper shows what changed in the PU prevention registration for the nurses after the introduction of DRAAI.

### 3.4 Context—At Baseline

The context at baseline was assessed through observation, reviewing current protocols and informal conversations with nurse champions and managers from each ward. Nurse champions are dedicated to PU prevention and wound care. They serve as primary contacts for ward nurses, provide education, address questions, identify problems, and monitor quality of care related to PU prevention and management. The context is described in detail in Table 1.

**TABLE 1:** Definitions, outcomes and data sources in this process evaluation based on the principles of Moore et al. (2015) in the pilot study of implementing DRAAI.

Process evaluation domain	Defined in this study as		
Context	Context at baseline Current PU screening—Until May 2023 the Waterlow Risk Score was required twice weekly and during any clinical changes. Since then, nurses have been encouraged to use their clinical reasoning to assess PU risk, with the Braden Scale available as backup in cases of uncertainty. For at‐risk patients, a nursing care plan must be created. This plan includes selecting preventive measures, which are automatically integrated into the nurses' activity overview in the electronic patient record. Additionally, it prompts nurses to document observations related to PU risk in a dedicated section that becomes visible if a care plan is created Medical beds—In May 2023, the hospital implemented pressure‐reducing mattresses. Previously, patients at risk for PU were transferred to beds with an alternating surface. Now, transfers are only necessary for patients at high risk for developing PU Nurse champions—Each ward has between one to seven nurse champions dedicated to PU prevention and wound care Daily stand‐up meetings—In the Covid‐19 and pulmonary wards, all nurses participate in daily stand‐up meetings. For internal medicine, the ward is divided into five zones, with one nurse from each zone attending the session. These sessions take place in the day shifts during weekdays Computer‐on‐wheels—All nurses have access to mobile desktop devices during their shifts, allowing them to consult the electronic patient record or access DRAAI via a web link as needed		
Complex intervention	Daily risk predictions in three risk categories: low risk, at‐risk or at high risk. The risk categories were shown in written text, by default only the at‐risk and at high‐risk patients are shown Stand‐alone web application in which the risk predictions were available Feedback on the follow‐up of the predictions (in line with hospital protocol) with creating a nursing care plan for at‐risk patients was incorporated Causal assumptions of DRAAI were: (1) daily risk predictions will reduce the feeling of registration burden among nurses, (2) attention towards PU prevention will increase and (3) this may result in timely pressure ulcer prevention		

Process evaluation domain	Defined in this study as	Outcome	Data source
Implementation	The process through which interventions are delivered, and what is delivered in practice.	Fidelity – % of admissions with at least one at‐(high)risk prediction that were followed up with creating a nursing care plan	Routine monitoring data
		Timeliness – % of admissions that receive preventive measures < 24 h	Routine monitoring data
		Feasibility of the implementation strategies	Field notes
		Adaptations–alterations made to DRAAI and the implementation in order to achieve better contextual fit	Field notes Updates DRAAI
		Dose – how many implementation strategies were delivered, for example, in number of visits by the research team	Field notes
Mechanism of impact	The intermediate mechanisms through which DRAAI results in intended (or unintended) effects. Nurses' responses – how participants interact with DRAAI	Acceptability – to assess if nurses had any concerns towards accepting DRAAI (trust, easy to consult, added value)	Questionnaire Field notes
		Feasibility – to assess potential challenges impacting the usability of DRAAI in practice (time, skills, fit with current work processes)	Questionnaire Field notes

Abbreviations: DRAAI, decubitus risk alert artificial intelligence; EPR, electronic patient record; NA, Not Applicable; PU, pressure ulcer.

### 3.5 Implementation

**FIGURE 2:**
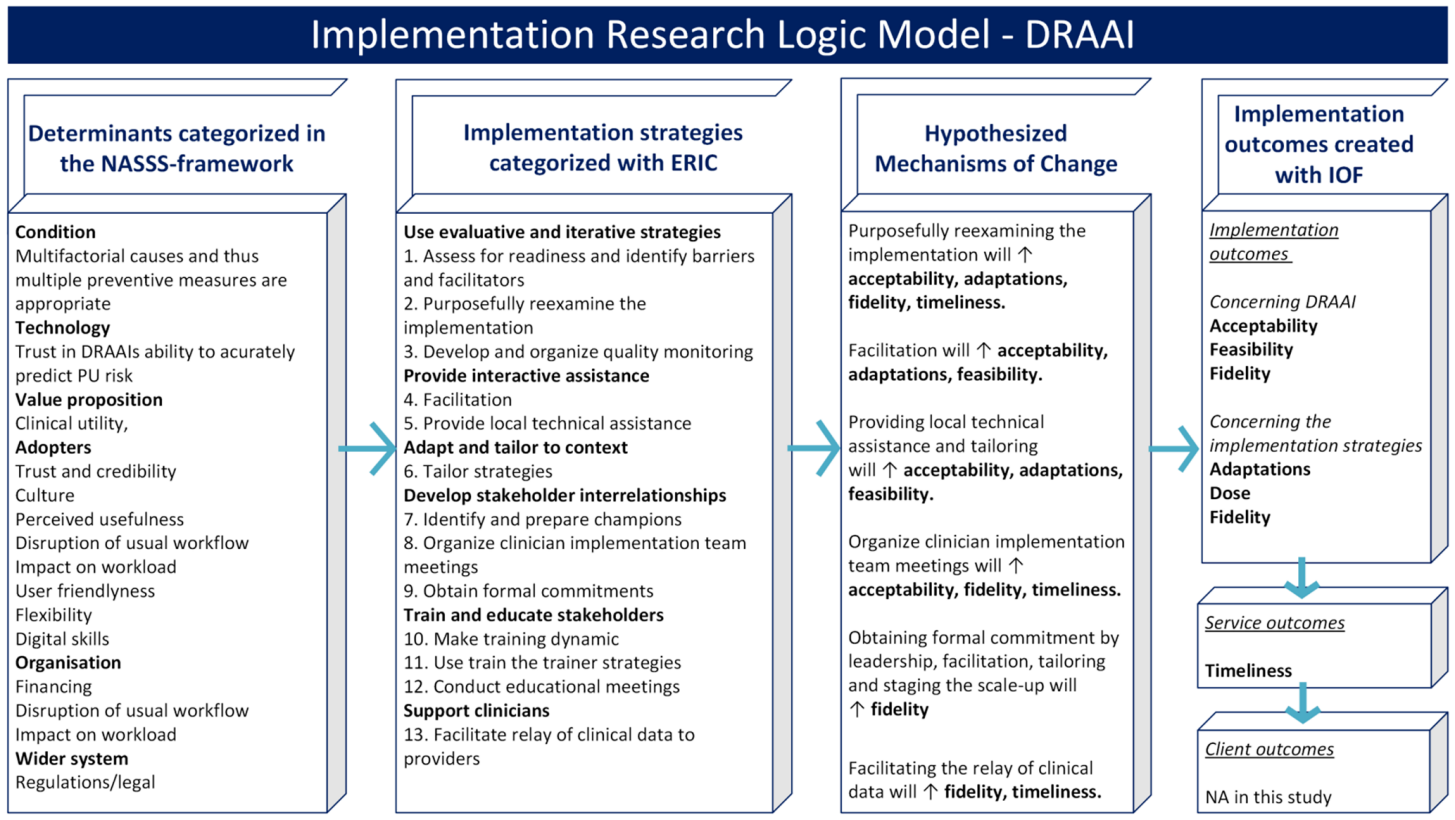
The implementation research logic model for DRAAI. NASSS, non‐adoption, abandonment, scale‐up, spread, sustainability (Greenhalgh and Abimbola 2019); ERIC, expert recommendations for implementing change (Waltz et al. 2015; Powell et al. 2015); IOF, implementation outcomes framework (Proctor et al. 2011); NA, not applicable. Blue Arrows indicate that these factors can influence the following steps: Specified definitions of the implementation outcomes are presented in Table 1. ↑ – increase. [Colour figure can be viewed at wileyonlinelibrary.com]

The Implementation Research Logic Model (IRLM) (Smith et al. 2020) guided the evaluation of DRAAI's implementation, examining the relationships between the intervention to determinants, strategies, mechanisms, and outcomes (Figure 2).

Determinants—barriers and facilitators—were categorised using the NASSS framework (Greenhalgh and Abimbola 2019). Determinants were identified by DS through a review of literature, particularly studies on implementing prediction models (Watson et al. 2020) and clinical decision support systems (Meunier et al. 2023). Additional determinants derived from clinical practice were added by project team members. The interdisciplinary project team included data scientists, a quality advisor with a nursing background, an implementation science PhD student with a nursing background, and a project manager. Key barriers included limited digital skills, lack of AI awareness, and concerns about trust and usability (Finkelstein et al. 2024; Araujo et al. 2020).

Thirteen implementation strategies were selected by the project team members—comprising data scientists, quality advisor, PhD student, project manager and nurse champions, nurse managers from selected wards—using the ERIC taxonomy (Waltz et al. 2015; Powell et al. 2015), based on their feasibility, acceptability, and expected impact. These strategies, spanning six ERIC domains, were operationalised following Proctor et al.'s guidelines (Proctor et al. 2013) (see Appendix S1). Local implementation teams (nurse champions, senior nurses, and managers) led the rollout, supported by the project team through educational sessions, daily stand‐ups, and ongoing technical assistance. In the first 2 weeks following the rollout of DRAAI on each ward, the project team actively facilitated the use of DRAAI while participating in the daily stand‐up meetings. Regular ward visits ensured continued support throughout the pilot. Nurse managers were asked to contribute to the implementation by formally stating their commitment to the importance of DRAAI being implemented and the prevention of PU. Just like for the champions, we asked them to articulate what their contributions would be during the pilot. They frequently reminded the team and asked how working with DRAAI and pressure ulcer prevention was going.

The hypothesised mechanisms of change (Figure 2) illustrate how strategies will adress barriers—for example, technical support for nurses with limited digital skills—will improve the implementation outcomes acceptability and feasibility.

Notably, the researchers and developers of DRAAI were actively involved in the implementation process.

### 3.6 Process Evaluation

Process evaluations aim to provide a comprehensive understanding to guide policy and practice. These evaluations typically cover five key aspects: context, intervention, implementation, mechanism of impact, and outcomes. In this process evaluation, we concentrated on context: examining how external factors, such as the reliability of the electronic patient documentation, affect the delivery and functioning of the interventions. Intervention: in which the intervention is described, including the causal assumptions about how the intervention will work. Implementation: exploring the structures, resources, and processes involved in the delivery of the intervention. This includes evaluating the implementation strategy, such as conducting educational sessions, and assessing both the quantity in number of sessions and quality in, for instance, the topics covered of what is delivered. Finally, the mechanisms of impact: investigating how the intervention and implementation strategies interact with the participants, and trigger changes (Moore et al. 2015). The outcomes are integrated within the previously mentioned aspects; all aspects are elaborated in the following paragraphs and summarised in Table 1.

### 3.7 Outcomes

The study outcomes were based on the Implementation Outcomes Framework by Proctor, Silmere (Proctor et al. 2011). The selected outcomes included acceptability, feasibility, and fidelity related to the implementation strategies. The evaluated implementation outcomes were adaptations, dose, and feasibility. Additionally, the service outcome of timeliness was assessed for the follow‐up of the predictions. Detailed definitions of these outcomes are provided in Table 1.

### 3.8 Data Collection

Three data sources were utilised. First, a questionnaire was administered to nurses to evaluate the acceptability and feasibility of DRAAI. A short questionnaire, developed by the project team members, included 30 questions based on the NASSS framework and the Unified Theory of Acceptance and Use of Technology (UTAUT) (Greenhalgh and Abimbola 2019; Marikyan 2023). It was made available online via LimeSurvey (LimeSurvey GmbH). To ensure participant anonymity, no demographic data were collected. The questionnaire was distributed from two up to 8 weeks after the rollout. Project team members encouraged nurses to complete it during informal ward visits and educational sessions. Nurses were eligible to participate if they worked on one of the participating wards, with no exclusion criteria applied.

Field notes were recorded by project team members, who were actively involved during the entire implementation period. These notes were stored in Siilo and a secure Microsoft Teams environment. All team members had smart phones, allowing them to document observations immediately following their visits. During the implementation, the frequency of joint daily stand‐up meetings, educational sessions, informal visits, evaluation meetings, and adaptations of the implementation strategies were collected. The number of nurses attending these sessions was also documented. Additionally, the team recorded the types of questions posed by nurses, how nurses responded to questions from the project team, and other noteworthy observations. The field notes provided insights into the mechanisms of impact. The project team also tracked the time invested in implementing the strategies, excluding preparation time.

During the study period, follow‐up of DRAAI risk predictions was assessed using routinely collected monitoring data. This was done by reviewing the nursing care plans of all admitted patients. The joint daily stand‐up meetings, which began at 07:45 AM, were chosen as the time when DRAAI predictions were presented to users. This timestamp was used in calculating the follow‐up time as ‘within X hours’. The follow‐up of risk predictions was automatically recorded for quality and control purposes. The follow‐up actions were categorised into four groups: (1) At‐risk according to nurse judgement prior to or without a prediction; (2) follow‐up within 24 h; (3) follow‐up after 24 h; and (4) no follow‐up. No demographic information or specific risk factors of the admitted patients were collected.

### 3.9 Data Analysis

To analyse the follow‐up of the predictions, the following approach was adopted. Since a single patient may have multiple admissions (e.g., a 3‐day admission in July and a 4‐day admission in September), we focused on admissions rather than individual patients. Quantitative data from questionnaires and field notes were analysed using descriptive statistics. Categorical variables are summarised as percentages. Statistical analyses were performed using IBM SPSS Statistics for Windows, Version 28.0. Armonk, NY: IBM Corp.

For the qualitative analysis of open‐ended responses and field notes, deductive content analysis was conducted. The implementation plan and the NASSS domains served as a framework for interpreting the data. The research team reviewed and discussed the field notes and open‐ended responses, identifying patterns, reflecting on the findings, and assigning meaning to the observations. A narrative summary of the main findings was incorporated into the results section where relevant (Elo and Kyngas 2008).

### 3.10 Ethical Considerations

Ethical considerations for retrieving data to generate and show the risk predictions adhered to hospital protocols, applicable privacy laws and regulations (Data Protection Impact Assessment) and principles for the responsible use of AI in healthcare (World Health Organization 2021). The Medical Ethics Review Committee of the Erasmus University Medical Center reviewed the study and determined that it was not subject to the Dutch Medical Research Involving Human Subjects Act. As a result, obtaining informed consent for this process evaluation study was deemed unnecessary (MEC‐2023‐0752).

## 4 Results

Overall, it was feasible to provide daily predictions for every patient throughout the entire pilot period. On two occasions, errors occurred during the preparation and display of the predictions; however, these issues were resolved within the same day.

### 4.1 Participants

A total of 55 nurses completed the questionnaire, resulting in a response rate of 39% (55/140). Of these, 25 nurses (43%; 25/58) were from the pulmonary ward, 21 (38%; 21/56) from the internal medicine ward, and nine (35%; 9/26) from the COVID‐19 ward.

### 4.2 Acceptability and Feasibility Among Nurses

Of the 55 nurses, 15 to 26 indicated in the questionnaire that they had not used DRAAI yet, with this number varying per question (see Table 2). Excluding the nurses that indicated they had not used DRAAI yet, most of the nurses agreed that DRAAI helps to assess the risk of developing a PU (88%; 35/40) and aids in PU prevention (92%; 35/38). Furthermore, most agreed that DRAAI is easy to consult (91%; 29/32). Many nurses preferred to consult DRAAI during day shifts, though five reported using it during evening shifts, and three during night shifts. Some nurses also noted discrepancies in PU risk estimation: 29% (16/55) felt the risk estimated by DRAAI was higher than their own assessment, while another 29% (16/55) felt it was lower. Notably, five nurses agreed with both statements.

**TABLE 2:** Nurses’ views on the value of DRAAI, the ease of access and agreement with the predicted risk (*N* = 55). [Colour table can be viewed at wileyonlinelibrary.com]

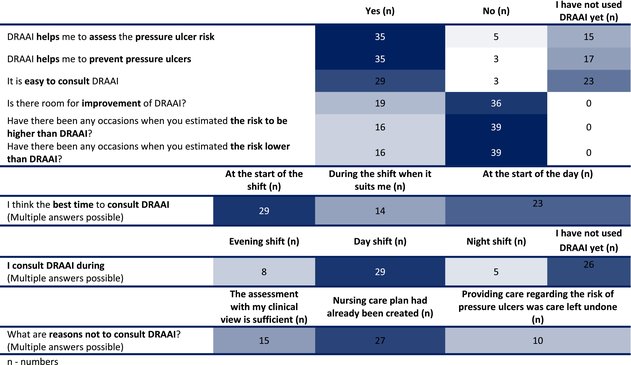

*Note:* The colour shades represent quartiles. < 25% is the lightest shade while the darkest shade represents > 75%.

Abbreviation: *n*, numbers.

A considerable number of nurses selected the neutral response option when asked about trusting DRAAI's predictions and whether their expectations were met, with 38% and 47% choosing neutral, respectively. On the other hand, the remaining nurses overall indicated that DRAAI's predictions largely met their expectations. Nearly half of the nurses (45%) expressed a desire for additional explanations on how DRAAI provides the risk predictions, see Table 3. Fieldnotes also frequently highlighted nurses' requests for up‐to‐date insights into the specific risk factors contributing to the predictions for each individual patient.

**TABLE 3:** Nurses’ perceptions on trust and follow‐up of DRAAI’s risk predictions. [Colour table can be viewed at wileyonlinelibrary.com]

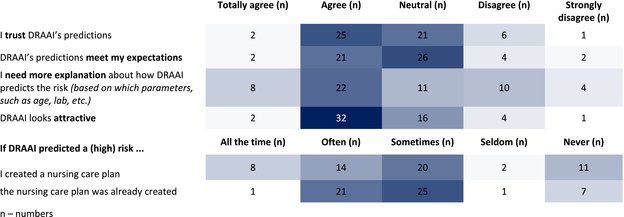

*Note:* The colour shades represent quartiles. < 25% is the lightest shade while the darkest shade represents > 75%.

Abbreviation: *n*, numbers.

Field notes revealed that nurses highly valued DRAAI's predictions as helpful reminders to perform PU prevention. Compared to the previously used Waterlow score, nurses noted that DRAAI was a significant improvement, particularly in reducing the registration burden. Several nurses provided feedback to enhance DRAAI, with most suggestions addressing practical issues, such as improving accessibility. One nurse raised the question about the relevance of gender being a risk predictor.

The use of a computer on wheels during the daily stand‐ups increased DRAAI's acceptability and fostered a joint understanding of the tool. Nurses began explaining DRAAI to one another, such as showing where the at‐risk patients were displayed. They also discussed individual patients flagged as at‐risk, using clinical reasoning to assess whether the predictions aligned with the patient's condition.

### 4.3 Dose of the Implementation Strategies

During the pilot, thirteen ERIC implementation strategies were employed, resulting in the following activities: four local implementation team meetings, thirty‐five stand‐up meetings, fifteen informal visits, and ten educational sessions. Implementing these strategies on the wards required approximately 1455 min in total. By counting session participants (rather than unique individuals), a cumulative attendance of 270 nurses was recorded. Among the strategies, attending all stand‐up meetings proved the most challenging to implement consistently, with a few instances where no project team members were present. One‐hundred‐and‐two unique users (73%; 102/140) accessed DRAAI's stand‐alone web page, collectively using the report 599 times.

A key implementation strategy was to *purposefully reexamine the implementation* to enhance the feasibility and fidelity of DRAAI's predictions by addressing practical issues. For example, the project team developed standardised content for discussion during the daily stand‐up meetings. However, in practice, the initial sessions focused more on the development, risk predictors, and reliability of DRAAI. This shift occurred partly due to nurses' questions and the need to build trust in the tool. Discussing specific cases with at‐risk predictions helped nurses better understand why a patient was classified as at‐risk for PU. By the second week, the focus shifted to correctly following up on risk predictions. This included selecting appropriate PU preventive measures, evaluating their effectiveness, and performing regular skin observations.

Another key implementation strategy involved *providing local technical assistance*. During the pilot, nurses required guidance on adding the web page to their favourites or creating a desktop shortcut, as it was accessed via a stand‐alone web application. Additionally, other important implementation strategies were *organising clinician implementation team meetings* and *conducting educational meetings*. All the strategies and their operationalization are described in Appendix S1.

### 4.4 Adaptations of DRAAI

The refinement of DRAAI during the pilot involved five updates to the dashboard visualisation and prediction model. The first update was necessary after the very first day, following feedback that the status of nursing care plans was not adequately displayed. This issue was addressed before the next day, fostering trust among nurses.

The second update added an information page to the dashboard, detailing all predictors used by the model and the direction of their effects. For example, ‘advanced age increases predicted risk.’

The third update introduced a slightly lower threshold for identifying at‐risk patients, based on nurses' feedback. Additionally, a horizontal bar was added to the dashboard to visually represent the predicted risk score.

During the fourth update, additional risk predictors were included. Nurses suggested several factors, such as patients receiving chemotherapy or using Optiflow, as indicators of elevated risk. While not all suggestions met the model's predictor requirements, some were incorporated, such as oxygen therapy and specific categories of antibiotics.

The fifth update during the pilot period included two major changes. The first change addressed feedback from the field notes. Nurses observed that patients who already had a PU continued to receive PU risk predictions. They emphasised the importance of displaying these patients separately, as they are inherently at risk of developing additional PUs. To resolve this, a new category ‘Pressure ulcer’ was added. Patients with an existing PU diagnosis were assigned to this category, and no further risk predictions were displayed for them. These patients were automatically shown alongside the at‐risk (high‐risk) patients. The second change stemmed from an issue identified by the project team and local implementation teams in usual care practices. Nursing care plans were being generated without including preventive measures, which made the real‐time feedback on prediction follow‐up incomplete. Initially, a green checkbox appeared when a PU nursing care plan was created. In the updated version of DRAAI, the green checkbox appeared only when a nursing care plan that included preventive measures was generated.

The project team informed the local implementation teams about the updates in person, via phone or mail, explaining the update and why they were performed.

The integration of the development and implementation teams proved advantageous for feasibility, as it allowed for adapting the intervention (DRAAI) and implementation strategies during the pilot phase, enhancing feasibility and responsiveness.

### 4.5 Adaptability and Feasibility of Implementation Strategies

The implementation strategies and adaptations were equally deployed across the three participating wards. During the implementation period, several adaptations were made to the implementation strategies to increase the feasibility of working with DRAAI. In this section, these adaptations are explained and justified. For one, we had not intended to share knowledge about current hospital protocols and PU prevention knowledge. Nevertheless, we became aware that nurses wished to alert the project team in cases when their clinical reasoning estimated a low risk for a patient to develop PU while DRAAI indicated a risk for developing PU.

It's annoying that the exclamation mark remains when I don't agree with the risk prediction.

However, when a member of the project team explained the PU risk, most of the nurses agreed that these patients actually had several risk factors. Some nurses believed DRAAI identified patients *with PU* rather than patients *with a risk of developing PU*. Thereupon, the project team clarified the difference between *risk predictions* and *PU detection* during the regular informal visits and educational sessions. Additionally, some nurses found it unnecessary to register PU risk for patients who had not yet developed PU.

Another adaptation was systematically addressing particular frequently asked questions (FAQs). These FAQs, along with detailed answers on how to address them, were distributed to the nurses via mail and, if possible, during educational sessions. The FAQs included the frequency of DRAAI's predictions, reasons for patients not receiving at‐risk predictions, and guidelines on preventive measures. Additionally, nurses expressed uncertainty about when they could discontinue preventive measures. However, DRAAI is designed to identify patients at high risk early in their admission, focusing on prompting nurses to initiate preventive measures rather than indicating when to stop them.

Nurses proposed changes to the nursing care plans in the electronic patient record, specifically requesting the automatic inclusion of preventive measures into the nursing activities and additional preventive measures, such as providing patient education or distributing educational material. Furthermore, the implementation of DRAAI sparked discussions about the essence of the nursing profession. Should PU prevention be an integral part of routine care? Do nurses truly need predictive tools to remind them, or should they be able to deliver this care independently?

What is the balance between thinking for oneself and receiving these pre‐chewed risk predictions at the beginning of your shift?

During the pilot period, we could not solve some feasibility issues but managed to address these issues a few weeks later. Examples include integrating DRAAI directly into electronic patient records and providing summaries of the risk factors contributing to the daily risk predictions for each patient. Such summaries could enhance nurses' understanding of the predictions and suggest potential preventive actions. Some nurses also envisioned a similar approach for other nurse‐sensitive outcomes in the future.

Nice to incorporate this [opening DRAAI and identifying patients at risk for developing a PU] into the joint daily stand‐ups; perhaps this is also suitable for future nurse sensitive outcomes.

### 4.6 Fidelity and Timeliness

All admissions during the 8‐week pilot period received daily updated risk predictions from DRAAI. A total of 428 admissions were included in the analysis to assess the follow‐up of the risk predictions, generating 2246 risk predictions. Among these, 128 admissions (30%) had at least one at‐risk prediction during their stay.

In terms of fidelity, nearly 80% (79%; 101/128) of the at‐risk predictions were followed up by nurses with the creation of a nursing care plan. Additionally, for 22% of the at‐risk predictions, a nursing care plan had already been created before DRAAI issued an at‐risk prediction. For instance, a patient admitted on August 3rd at 15:00 might have had a nursing care plan created at 17:00, while the first DRAAI prediction on the morning of August 4th flagged the patient at risk. In 72% (21/29) of such cases, the first DRAAI prediction for the admission flagged at risk as well.

Timeliness of follow‐up showed that for 65% of patients, a nursing care plan was created within 48 h of the first at‐risk prediction, as shown in Table 4.

**TABLE 4:** Fidelity and timeliness concerning the follow‐up of the risk predictions.

Description of the outcome	Number of admissions	Percentage
Number of admissions that received daily predictions from DRAAI	428	100
**Fidelity**		
Number of admissions in which there was at least one at (high) risk prediction from DRAAI	128	100
A nursing care plan was created	101	78.9
Number of admissions where there was never at (high) risk in DRAAI	300	100
No at (high) risk prediction from DRAAI, a nursing care plan was created based on clinical reasoning/other risk assessment was performed	43	14.3
**Timeliness**	128	100
Nursing care plan created prior to at (high) risk prediction from DRAAI	29	22.7
Nursing care plan created within 24 h of at (high) risk prediction from DRAAI	40	31.3
Nursing care plan created between 24 and 48 h of first at (high) risk prediction from DRAAI	14	10.9
Nursing care plan created later than 48 h of first at (high) risk prediction from DRAAI	18	14.1
Never created a nursing care plan	27	21.0

## 5 Discussion

The pilot implementation of DRAAI was well‐received among nurses, demonstrating its potential to enhance PU prevention. Most nurses found DRAAI predictions helpful, particularly for early risk assessment and reducing the registration burden compared to the Waterlow score. However, the need for clearer communication regarding the distinction between PU risk prediction and PU detection, as well as additional explanations on how DRAAI generates its predictions, became apparent. Concurrently, a notable portion expressed neutrality or uncertainty about its predictions and ease of use. The implementation strategies were generally feasible, though attending all daily stand‐up meetings was challenging for the project team. Continuous evaluation during the pilot phase led to several adaptations to both DRAAI and the implementation strategies. These adjustments were crucial for addressing practical challenges and improving DRAAI's usability. Overall, high fidelity in follow‐up actions for PU prevention was observed, with most of the at‐risk predictions resulting in timely preventive PU measures. This pilot underscores the importance of ongoing engagement, proactive adaptations to the implementation plan, and iterative refinements to fully integrate DRAAI into clinical practice. Ultimately, these efforts aim to better support nurses in delivering improved patient care.

Integrating AI‐based prediction models into daily practice can potentially assist nurses in clinical decision‐making, enabling more evidence‐based and personalised care (Maleki Varnosfaderani and Forouzanfar 2024; Ronquillo et al. 2021). Nevertheless, significant challenges remain in successfully integrating AI into healthcare. Ronquillo, Peltonen (Ronquillo et al. 2021) conducted an international think‐tank and identified three key priorities for AI integration: (a) Nurses must understand the relationship between the data they collect and the AI technologies they use; (b) Nurses should be actively involved in all stages of AI development and implementation; and (c) there is substantial untapped potential for nursing to contribute to the development of AI technologies for global health and humanitarian efforts.

Our study addressed the first two topics during the development and implementation phases of DRAAI. In the development phase, we co‐developed DRAAI with nurse champions and wound care nurses, which provided valuable insights into nursing practice and the quality of nurses' documentation in patient records regarding PU risk (Greup et al. 2024).

Several nurses indicated that they had not used DRAAI yet; we excluded these responses from the analysis where nurses were able to indicate this. However, we believe they may have interpreted the question differently than we intended. It was not necessary that all nurses individually assessed the weblink to DRAAI, as the dashboard is typically reviewed collectively during the joint daily stand‐up meetings, where patients requiring extra care or attention are discussed. Interestingly, many of the nurses who reported not having used DRAAI still provided valuable suggestions for improvement—suggesting that they had, in fact, interacted with the tool in some capacity.

During the implementation phase, we engaged in several discussions about the predictions that nurses disagreed with. This may impact building trust among healthcare professionals in AI for clinical decision‐making. Which is a gradual process (Meskó and Görög 2020), it is, therefore, not surprising that nurses were relatively neutral about trusting DRAAI's predictions during this three‐month pilot study. Many instances of disagreement with the risk predictions stemmed from a misunderstanding of its purpose—specifically, the distinction between *risk predictions* and *PU detection*. Clarifying that DRAAI was designed to support nurses in assessing *PU risk*, rather than *detecting PU*, resolved much of this confusion. Additionally, the perception of PU risk as a minor issue among some nurses may reflect gaps in knowledge, attitude, or a sense of responsibility in PU prevention (Li et al. 2022). Many nurses also viewed the creation of nursing care plans for at‐risk patients as a registration burden. This raises concerns about whether nurses fully understand the value and interrelation of nursing diagnoses, care goals, interventions, and evaluations within care plans (Rossi et al. 2023). Another potential factor is the sufficiency of healthcare literacy among nurses (Toronto 2016). It is important to note that fully trusting AI for clinical decision‐making carries inherent risks, as the accuracy of risk predictions relies heavily on the input data (Abell et al. 2023). Reassuringly, in this pilot study, nurses identified 14% of patients as being at high risk for PUs based on their clinical judgement, even though DRAAI had not yet flagged them as such. This highlights the importance of balancing trust in AI with the critical judgement of nurses.

Incorporating implementation science principles in this pilot study facilitated a systematic approach to identifying and addressing key determinants, developing an implementation plan, and combining multiple models, frameworks, and theories. This approach enabled the adaptation of both the intervention and its implementation strategies, ultimately enhancing their effectiveness and success.

Determinants continued to emerge during the implementation, for instance the misalignments of practice with hospital protocol. The pilot allowed for adjustments to address these determinants effectively. While the proposed implementation strategies and adaptations were deemed time‐consuming, they were deemed feasible by the project team and the local implementation teams. For instance, the project team's daily presence during joint stand‐ups in the first 2 weeks was particularly time‐consuming, especially given the sequential aspect of this pilot. This required the project team to attend daily stand‐ups over 6 weeks since it was not feasible to attend multiple wards simultaneously, as most stand‐ups occurred at the same time.

The need for adaptability of interventions and implementation strategies has gained increasing attention in research, including the design of adaptive trials (Pallmann et al. 2018) and methods to track adaptations in implementation strategies (Holtrop et al. 2022). The project team played an active role in communicating findings and adapting both the intervention and its implementation in response to determinants. These adaptations were carefully documented in field notes.

The accessibility of DRAAI within the electronic health record was an important barrier, with nurses frequently asking how they could access it more easily. Integrating DRAAI within the electronic patient record minimises disruptions of nurses' workflow, thereby enhancing the acceptability and feasibility of using a clinical decision support system (Meunier et al. 2023).

## 6 Strengths and Limitations

A significant strength of our study was the collaboration among nursing scientists, data scientists, quality and care teams, and health care professionals. From the development phase through the evaluation phase, close collaboration with data scientists facilitated rapid updates and data checks, allowing identified issues to be resolved within a single day. He et al. (2019) highlights the critical role of such collaboration in ensuring the success of AI implementations in healthcare. The involvement of quality and care teams and nursing scientists fostered alignment between clinical practice and science.

Another important strength was the collaborative and adaptive nature of the model's refinement process. By inviting nurses to suggest additional predictors, we not only encouraged active participation but also ensured that the model reflected clinical insights from diverse wards. This inclusive approach helped surface potentially overlooked data points—particularly from internal medicine wards that were not represented during the initial development phase. Importantly, any suggested additions were not automatically incorporated into the model. Each proposed element was reviewed by the expert group to assess whether it was clinically explainable and potentially valuable. Only those predictors that met these criteria were considered for further investigation, and a few were ultimately integrated into the model. This approach allowed us to balance evidence‐based development with practical, clinical insights, ensuring the model remained both scientifically grounded and contextually relevant.

However, the involvement of the project team also presented certain limitations. While their embedded role enabled rapid adjustments to implementation strategies, it may have compromised objectivity and introduced the risk of social desirability bias (Graeff 2005). Additionally, the short interval between distributing the questionnaire and nurses' exposure to DRAAI—ranging from 2 to 8 weeks—may have contributed to inconsistent responses. Some nurses reported not having used DRAAI themselves, though they may have encountered it during daily stand‐ups or while observing colleagues.

We conducted extensive evaluation sessions with the local implementation teams; however, these sessions were not recorded. Relying solely on field notes, without supplementing them with additional interviews or focus group discussions, limited our ability to gain a deeper understanding of the mechanisms of change that were addressed or overlooked in this pilot study.

Another limitation of this study is that we did not collect demographic data such as educational level and frequency of using digital technologies from the nurses involved in our study. A cross‐sectional survey found that nurses' digital health literacy is strongly influenced by education level, professional role, habits and attitude towards digital technologies (Comparcini et al. 2024). Future studies should consider collecting these demographics, especially since the digital skills among nurses were an important barrier and enabler for using DRAAI.

## 7 Recommendations

A unique aspect of this study is the implementation of an AI‐based prediction model in clinical practice, unlike many other models that fail to progress beyond the development phase. This success is largely due to our continuous re‐examination of the implementation process. Without this ongoing evaluation, DRAAI would probably have failed. Therefore, our key recommendations include performing continuous re‐examination of the implementation process, ethical aspects, quality control, and involving patients.

Continuous re‐examination of the implementation process ensured us to detect that nurses felt that DRAAI's risk predictions were unreliable. This experience underscores the critical importance of nurses' understanding and trust in AI. As this is the first AI risk prediction model implemented in practice for nurses, it raises the question whether they will maintain this level of scrutiny towards future AI models. On the other hand, it remains essential to consider ethical aspects, rigorously perform quality control checks of AI technologies in healthcare (Inglada Galiana et al. 2024; Keskinbora 2019). Especially when relying on AI becomes more relevant.

As AI systems become more integrated into healthcare, one potential strategy to enhance trust is to involve patients. By sharing AI‐generated predictions with patients, we might be able to foster greater patient engagement and participation in taking measures to prevent the development of PU (Latimer et al. 2014). This approach may not only empower patients but also promote a collaborative environment where both healthcare providers and patients work together towards better health outcomes.

Before scaling‐up DRAAI to other hospitals we propose a few recommendations for future research. First, a rigorous investigation focusing on the effectiveness of DRAAI in reducing PU incidence. Secondly, investigating the intensive implementation strategies, for example, facilitating the daily stand‐ups need to be revisited and adapted to settings with fewer resources or staff. We are currently performing a hybrid type 2 study (Curran et al. 2012) in the same university hospital which simultaneously examines the effectiveness of the clinical decision support tool DRAAI and the effects of the implementation strategies. In preparation of this study, we continued to improve the visualisation of the dashboard and the contents, making it possible to provide insights in the contribution of each risk predictor per patient and the predicted risks over time. Additionally, we recommend considering using AI for designing educational materials and designing and selecting implementation methods and strategies (Younas and Reynolds 2024).

## 8 Conclusion

This pilot implementation study gave us confidence that implementing an AI‐based prediction model to identify patients at risk for developing PUs is both acceptable and feasible. Nurses responded positively to the model and the implementation plan, which was supported by continuous evaluation, intensive facilitation, daily reminders, and formal commitment. These were key factors in the successful implementation of DRAAI. The study also found that nurses generally adhered to DRAAI's risk alerts, showing good fidelity, and that follow‐up actions were taken promptly. These findings suggest that DRAAI has the potential to improve PU prevention. Future research should evaluate the model's clinical impact and effectiveness in reducing PU incidence. Ultimately, integrating AI‐driven clinical decision support tools like DRAAI into routine nursing practice could help nurses provide more effective PU prevention care.

## Author Contributions

D.S. created the study concept and design. D.S., A.V., M.D., S.G. and E.I. provided feedback on the design. D.S. and A.V. were actively involved in the implementation and collected data along the process. D.S. completed the data analysis and interpretation of the data. The qualitative data was discussed with D.S. and A.V. D.S. was responsible for drafting the manuscript and writing the final report and is the guarantor for the paper. A.V., M.D., S.G. and E.I. performed critical revision of the contents. D.S., A.V., M.D., S.G. and E.I. read and approved the final manuscript.

## Funding

The authors have nothing to report.

## Ethics Statement

The ethical considerations for retrieving and generating the risk predictions followed hospital protocol, current laws and regulations concerning privacy (DPIA) and using AI in healthcare responsibly [44]. The Medical Ethics Review Committee of the Erasmus University Medical Center assessed this study as not subject to the Dutch Medical Research Involving Human Subjects Act and confirmed that gathering informed consent for this process evaluation study was not necessary (MEC‐2023‐0752).

## Consent

All authors have agreed with the contents of the manuscript to be published.

## Conflicts of Interest

The authors declare no conflicts of interest.

## Supporting information


**Appendix S1:** jocn70212‐sup‐0001‐AppendixS1.docx.

**Appendix S2:** jocn70212‐sup‐0002‐AppendixS2.docx.

**Appendix S3:** jocn70212‐sup‐0003‐AppendixS3.pdf.

## Data Availability

The data that support the findings of this study are available on request from the corresponding author. The data are not publicly available due to privacy or ethical restrictions.
