# Supplementary material for: Integrating Artificial Intelligence in Nursing Practice With Decubitus Risk Prediction Alerts: A Pilot Process Evaluation
Source: J Clin Nurs. 2026 Jan 20;35(10):4184–97. doi: 10.1111/jocn.70212 (PMC13569127; doi:10.1111/jocn.70212)
Supplement: Supplementary file 1 [file JOCN-35-4184-s001.pdf]

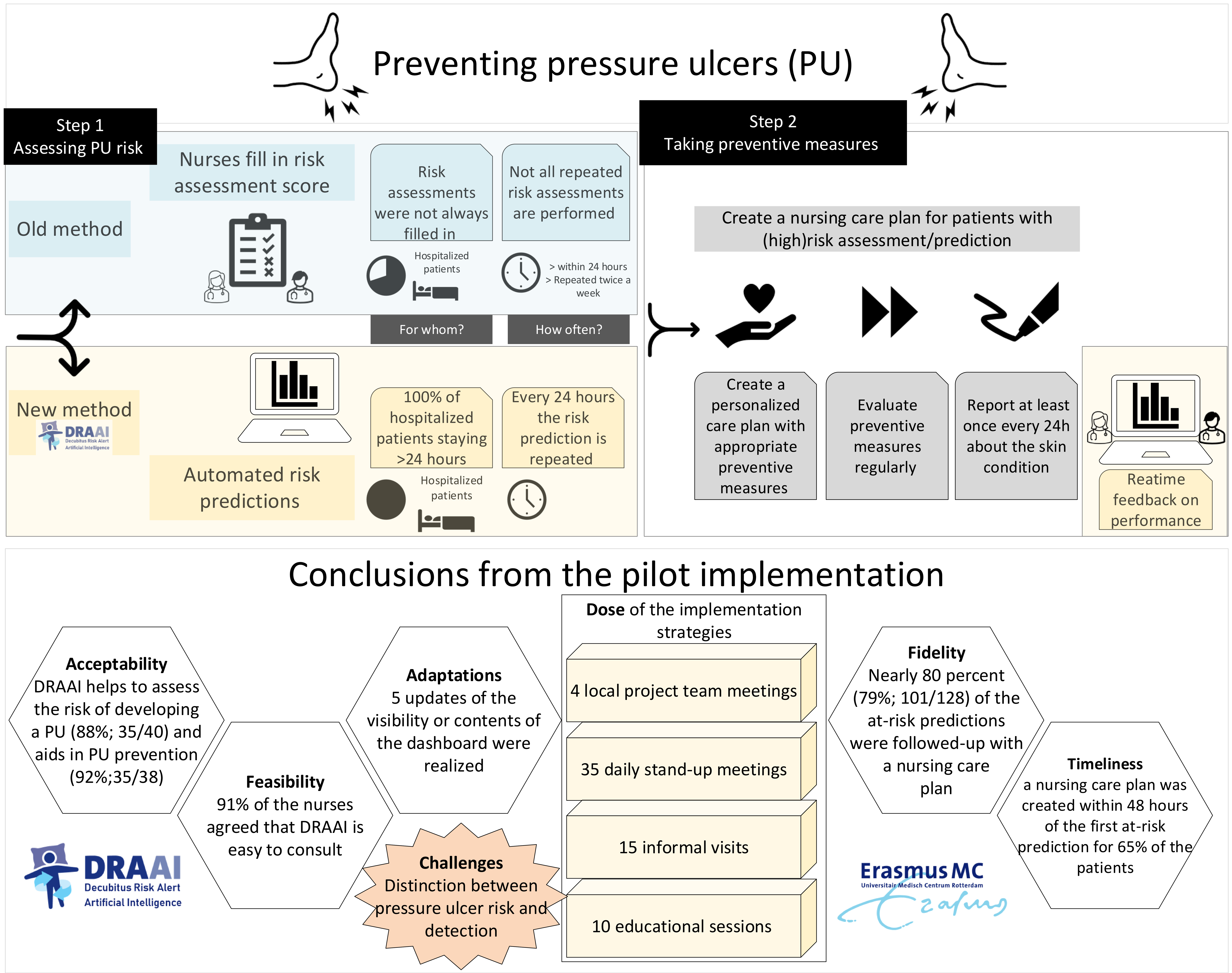
Preventing pressure ulcers (PU)
Step 1
Assessing PU risk
Old method
Nurses fill in risk assessment score
Risk assessments were not always filled in
Not all repeated risk assessments are performed
Hospitalized patients
> within 24 hours
> Repeated twice a week
For whom?
How often?
New method
DRAAI
Automated risk predictions
100% of hospitalized patients staying >24 hours
Every 24 hours the risk prediction is repeated
Hospitalized patients
Step 2
Taking preventive measures
Create a nursing care plan for patients with (high)risk assessment/prediction
Create a personalized care plan with appropriate preventive measures
Evaluate preventive measures regularly
Report at least once every 24h about the skin condition
Reatime feedback on performance
Conclusions from the pilot implementation
Acceptability
DRAAI helps to assess the risk of developing a PU (88%; 35/40) and aids in PU prevention (92%;35/38)
Feasibility
91% of the nurses agreed that DRAAI is easy to consult
Adaptations
5 updates of the visibility or contents of the dashboard were realized
Challenges
Distinction between pressure ulcer risk and detection
Dose of the implementation strategies
4 local project team meetings
35 daily stand-up meetings
15 informal visits
10 educational sessions
Fidelity
Nearly 80 percent (79%; 101/128) of the at-risk predictions were followed-up with a nursing care plan
Timeliness
a nursing care plan was created within 48 hours of the first at-risk prediction for 65% of the patients
DRAAI
Decubitus Risk Alert
Artificial Intelligence
Erasmus MC
