# Supplementary material for: Integrating Artificial Intelligence in Nursing Practice With Decubitus Risk Prediction Alerts: A Pilot Process Evaluation
Source: J Clin Nurs. 2026 Jan 20;35(10):4184–97. doi: 10.1111/jocn.70212 (PMC13569127; doi:10.1111/jocn.70212)
Supplement: Supplementary file 3 [file JOCN-35-4184-s003.docx]

Supplemental file 1 - Implementation strategies

| **Implementation strategy as defined by ERIC** | **From ERIC Domain** | **Actor** | **Action** | **Action Target** | **Dose** | **Temporality** | **Implementation outcome affected** | **Justification** |
| --- | --- | --- | --- | --- | --- | --- | --- | --- |
| **1. Assess for readiness and identify barriers and facilitators** | Use evaluative and iterative strategies | Project group, together with local implementation teams | Assessing barriers and facilitators from field notes, informal conversations, educational sessions, short surveys | Nurses, nursing assistants, nurse managers | 15 informal visits performed (5 up to 30 minutes per ward)  10 educational sessions  55 responses were collected through a survey | Informal conversations continuously.  Survey during the pilot. Between 3-8 weeks after the start. | Acceptability, feasibility | The NASSS-Framework was used to categorize the barriers identified from literature and clinical expertise. Barriers were extracted from the literature, extracted from papers implementing prediction models (1) or decision support systems (2). Barriers from previous implementation efforts in our hospital, which were gathered in questionnaires and interviews after the implementation of smart medical beds. |
| **2. Purposefully reexamine the implementation** | Use evaluative and iterative strategies | Project group | - Repeated evaluations within the project team, as well as repeated (in)formal evaluations with PU nurses, senior nurses and nurse managers throughout the entire period. - One evaluation meeting was performed with the project group and local implementation team for the Internal Medicine ward, the other evaluation meeting was performed with the Pulmonary and COVID-19 ward together. - If needed the implementation strategies changed slightly, e.g. more visits to provide interactive assistance or an extra educational session | Align with changes in the context, barriers and facilitators and needs of target group | - Evaluation within the project team lasted 5 up to 30 minutes. - Informal evaluations lasted 2 up to 30 minutes with individuals or small groups of nurses/PU nurses/Nurse managers - Formal evaluation took about 30 up to 60 minutes. | - Project team evaluations were conducted frequently throughout the pilot period - Informal evaluations were conducted frequently throughout the pilot period - Formal evaluations were planned between 5 and 8 weeks after the start of the pilot | Acceptability, Feasibility | To address the determinants *Disruption of usual workflow, flexibility* and *Impact on workload,* |
| **3. Develop and organize quality monitoring** | Use evaluative and iterative strategies | Project group | Ensure real-time feedback on the follow-up of the risk prediction.  Continuously we improved the visualization of the feedback according to the input from nurses, in one of the updates the nursing care plan pressure ulcer was added, next to the nursing care plan at risk for pressure ulcers. | Nurses, nurse champions, nurse managers, | Every 15-minutes the follow-up of the risk predictions was updated according to the actions taken by nurses (create a nursing care plan) | Realized from the start of the pilot, and improved in an update during the pilot | Fidelity | To address the determinants *Clinical utility* and U*ser friendliness* |
| **4. Facilitation** | Provide interactive assistance  Provide interactive assistance | Project group, senior nurses, nurse managers | Visit the joint daily sessions. | Nurses and nursing assistants, nurse managers | Lasting between 2 and 10 minutes | Daily visits for two weeks after the introduction of DRAAI | Adoption | To address the determinants *Clinical utility, Flexibility, Trust and credibility*, *Perceived usefulness,* and U*ser friendliness* |
|  |  | Project group | Visit the wards during slow hours. informally asking questions about working with DRAAI, challenges they are experiencing, why they are or are not working with it. | Nurses and nursing assistants that are sitting behind a desk, drinking coffee or are relaxing. | Lasting between 2 and 20 minutes | 15 informal visits were conducted, see timeline visual | Adoption | To address the determinants *Clinical utility, Trust and credibility*, *Perceived usefulness,* and U*ser friendliness* |
| **5. Provide local technical assistance** | Provide interactive assistance | project group | Visit the wards during slow hours. informally asking to show where to find DRAAI, how to perform appropriate follow-up, etc. | Nurses and nursing assistants that are sitting behind a desk, drinking coffee or are relaxing. | Lasting between 2 and 20 minutes | 15 informal visits were conducted, see timeline visual | Acceptability, feasibility | To address the determinants *Clinical utility, Trust and credibility*, *Perceived usefulness,* and U*ser friendliness* |
| **6. Tailor strategies** | Adapt and tailor to context | Project group, together with local implementation teams | In collaboration the implementation strategies were carefully selected and tailored depending on the culture within the ward, current work processes and workflow. | Nurses, nursing assistants, nurse champions, nurse managers | The implementation strategies were tailored during the kick-off meeting, and according to the input from the informal visits |  | Acceptability, feasibility | To address the determinants *Culture*, *Clinical utility, Trust and credibility*, *Perceived usefulness,* and *User friendliness* |
| **7. Identify and prepare champions** | Develop stakeholder inter-relationships | Project group, nurse managers | The project team asked the nurse champions to participate in the local implementation teams. These nurse champions were already champions in wound care and pressure ulcer prevention, during the local implementation team meetings the nurse champions were prepared for their role as a champion for DRAAI. The nurse champions were asked to articulate what their contributions would be during the pilot. They frequently reminded the team, asked around for feedback and supported colleagues in using DRAAI as intended | Nurse champions | The first local implementation team meeting took about 30 up to 60 minutes. | At least one week before the kick-off of DRAAI in the ward | Fidelity, sustainability | To address the determinants *Trust and credibility, Trust in DRAAIs ability to accurately predict PU risk,* and *Culture* |
| **8. Organize clinician implementation team meetings** | Develop stakeholder inter-relationships | Project group, together with local implementation teams | The project team asked the nurse managers and the nurse champions to participate in the local implementation teams. The local implementation teams ensured close monitoring of the wards. They quickly identified whether changes were needed to DRAAI itself, which questions were frequently asked, or if adjustments to the implementation strategy were necessary, for example, in the form of an additional educational session. | Nurse champions | - The first local implementation team meeting took about 30 up to 60 minutes. - Formal evaluation took about 30 up to 60 minutes. | At least one week before the kick-off of DRAAI in the ward was the first implementation team meeting.  The evaluation meeting | Acceptability, feasibility | To address the determinants *Disruption of usual workflow*, and *Impact on workload* |
| **9. Obtain formal commitments** | Develop stakeholder inter-relationships | Project group | The project team asked the nurse managers to participate in the local implementation teams. During the local implementation team meetings, the nurse managers were prepared for their role in the implementation of DRAAI. The nurse managers were asked to contribute to the implementation by formally stating their commitment to the importance of DRAAI being implemented and the prevention of PU. Just like for the champions, we asked them to articulate what their contributions would be during the pilot. They frequently reminded the team, and asked how working with DRAAI and pressure ulcer prevention was going | Nurse managers | - The first local implementation team meeting took about 30 up to 60 minutes. | - At least one week before the kick-off of DRAAI in the ward - Informal evaluations were conducted frequently throughout the pilot period - Formal evaluations were planned between 5 and 8 weeks after the start of the pilot | Adoption | To address the determinants *impact on workload, Culture, Disruption of usual workflow* |
| **10. Make training dynamic** | Train and educate stakeholders | Project group, nurse champions | Educational sessions were interactive and dynamic. At least one member of the project team participated and a member of the local implementation team. For the second and third ward we invited nurses from the wards that already started with DRAAI to explain how it works and how it helped them, but also which challenges had to be overcome.  During the training nurses could practice with exploring the dashboard, such as the risk factors that the model includes, but also with creating nursing care plans in the right way. The value of using nursing care plans, and what should be reported in which part of the electronic patient file (skin observation, preventive measures, etc.) | Nurses, nursing assistants | Lasting about 20 up to 45 minutes.  The content focused on the risk factors of DRAAI, nursing care plans, the concept of being at risk, clinical reasoning in PU prevention and PU prevention in general | Ten educational sessions were performed during the 8-week pilot period | Acceptability, feasibility, sustainability | To address the determinants *Clinical utility, Trust and credibility*, *Perceived usefulness,* and *User friendliness* |
| **11. Use train the trainer strategies** | Train and educate stakeholders | Project group, together with local implementation teams | The members of the local implementation teams were asked to participate in at least one of the educational sessions. After the educational session the project team member discussed the educational session and inquired if the local implementation team member would be prepared to conduct educational sessions by themselves or if they needed additional guidance. If so, an additional appointment was planned. | Nurse champions | Nurse champions were prepared during the educational sessions which lasted between 20 and 45 minutes. All nurse champions felt prepared after participating in one or two education sessions. | Ten educational sessions were performed during the 8-week pilot period. | Penetration, acceptability | To prepare for sustainability |
| **12. Conduct educational meetings** | Train and educate stakeholders | Project group, together with local implementation teams | See strategy 10. | Nurses, nursing assistants | See strategy 10. | See strategy 10. | Fidelity | To address the determinants *Clinical utility, Trust in technology*, *Perceived usefulness,* and U*ser friendliness* |
| **13. Facilitate relay of clinical data to providers** | Support clinicians | Project group | DRAAIs dashboard provides a visualization of the risk predictions per patient as well as the follow-up of the risk predictions with nursing care plans.  After one of the evaluation meetings, nurses suggested that not only the nursing care plan at risk for Pressure Ulcers should be in DRAAI but also the nursing care plan Pressure Ulcer. With this addition, nurses, nursing assistants and nurse managers receive an overview of the number of admitted patients, the number of patients at (high) risk and the number of patients with a pressure ulcer | Nurses, nursing assistants, nurse champions, nurse managers | Daily update of the risk prediction (5 AM)  Real-time feedback on the follow-up of the risk predictions with nursing care plans (every 15 minutes) | Continuously | Fidelity | To address the determinants *Clinical utility, Perceived usefulness,* and U*ser friendliness* |

Abbreviation: ERIC – Expert Recommendations for Implementing Change

1.	Watson J, Hutyra CA, Clancy SM, Chandiramani A, Bedoya A, Ilangovan K, et al. Overcoming barriers to the adoption and implementation of predictive modeling and machine learning in clinical care: what can we learn from US academic medical centers? JAMIA Open. 2020;3(2):167-72.

2.	Meunier PY, Raynaud C, Guimaraes E, Gueyffier F, Letrilliart L. Barriers and Facilitators to the Use of Clinical Decision Support Systems in Primary Care: A Mixed-Methods Systematic Review. Ann Fam Med. 2023;21(1):57-69.
